# Incremental prognostic value of ADC histogram analysis in patients with high-risk prostate cancer receiving adjuvant hormonal therapy after radical prostatectomy

**DOI:** 10.3389/fonc.2023.1076400

**Published:** 2023-01-25

**Authors:** Kangwen He, Yucong Zhang, Shichao Li, Guanjie Yuan, Ping Liang, Qingpeng Zhang, Qingguo Xie, Peng Xiao, Heng Li, Xiaoyan Meng, Zhen Li

**Affiliations:** ^1^ Department of Radiology, Tongji Hospital, Tongji Medical College, Huazhong University of Science and Technology, Wuhan, China; ^2^ Department of Geriatrics, Tongji Hospital of Tongji Medical College, Huazhong University of Science and Technology, Wuhan, China; ^3^ School of Data Science, City University of Hong Kong, Hong Kong, Hong Kong SAR, China; ^4^ College of Life Science and Technology, Huazhong University of Science and Technology, Wuhan, China; ^5^ Department of Urology, Tongji Hospital, Tongji Medical College, Huazhong University of Science and Technology, Wuhan, China

**Keywords:** high-risk prostate cancer, biochemical recurrence, magnetic resonance imaging, apparent diffusion coefficient, histogram analysis

## Abstract

**Purpose:**

To investigate the incremental prognostic value of preoperative apparent diffusion coefficient (ADC) histogram analysis in patients with high-risk prostate cancer (PCa) who received adjuvant hormonal therapy (AHT) after radical prostatectomy (RP).

**Methods:**

Sixty-two PCa patients in line with the criteria were enrolled in this study. The 10^th^, 50^th^, and 90^th^ percentiles of ADC (ADC_10_, ADC_50_, ADC_90_), the mean value of ADC (ADC_mean_), kurtosis, and skewness were obtained from the whole-lesion ADC histogram. The Kaplan–Meier method and Cox regression analysis were used to analyze the relationship between biochemical recurrence-free survival (BCR-fs) and ADC parameters and other clinicopathological factors. Prognostic models were constructed with and without ADC parameters.

**Results:**

The median follow-up time was 53.4 months (range, 41.1-79.3 months). BCR was found in 19 (30.6%) patients. Kaplan−Meier curves showed that lower ADC_mean_, ADC_10_, ADC_50_, and ADC_90_ and higher kurtosis could predict poorer BCR-fs (all *p*<0.05). After adjusting for clinical parameters, ADC_50_ and kurtosis remained independent prognostic factors for BCR-fs (HR: 0.172, 95% CI: 0.055-0.541, *p*=0.003; HR: 7.058, 95% CI: 2.288-21.773, *p*=0.001, respectively). By adding ADC parameters to the clinical model, the C index and diagnostic accuracy for the 24- and 36-month BCR-fs were improved.

**Conclusion:**

ADC histogram analysis has incremental prognostic value in patients with high-risk PCa who received AHT after RP. Combining ADC_50_, kurtosis and clinical parameters can improve the accuracy of BCR-fs prediction.

## 1 Introduction

Prostate cancer (PCa) is the second most common cancer and fifth leading cause of cancer-related deaths in men worldwide, with an estimated nearly 1.4 million new cases and 375,000 deaths in 2020 (1). For men with high-risk localized and locally advanced PCa, multimodal therapy strategies, such as a reasonable first step of radical prostatectomy (RP) to reduce the tumor burden, followed by adjuvant androgen deprivation therapy or radiation therapy, are often necessary (2). Several studies have found considerable benefits for patients receiving adjuvant hormonal therapy (AHT) after RP compared to observations after RP (3–6). However, the current definition of high-risk prostate cancer includes a heterogeneous group of patients with different outcomes based on different baseline characteristics (7). A proportion of patients may exhibit early resistance to AHT and elevated PSA levels in the short term after RP, which has given rise to studies of prognostic factors, including novel biomarkers such as advanced magnetic resonance imaging (MRI) parameters.

In recent years, MRI has been widely used in clinical practice for the detection and staging of PCa (8) and is also promising in predicting the prognosis of PCa (9, 10). The apparent diffusion coefficient (ADC), derived from diffusion-weighted MRI, is thought to be related to the cellularity and interstitial structure of pathological tissue and therefore reflects the histopathological heterogeneity of malignant tumors (11–13). Histogram analysis is a texture-based statistical measurement method for images that can quantitatively provide a series of information on ADC maps, such as percentile ADC values, kurtosis, and skewness. Numerous studies have shown that ADC histogram analysis derived from baseline MRI examination has prognostic value in several types of tumors, including glioblastoma (14), esophageal carcinoma (15), and squamous cell cervical cancer (16). In PCa, ADC histogram analysis has recently been declared to be valuable in the detection, differentiation, and aggressiveness assessment of cancer lesions. Peng et al. (17) found that ADC_10_ and ADC_mean_ could differentiate between prostate cancer and normal tissue. Donati et al. (18) evaluated the correlation between the 10^th^ percentile, 25^th^ percentile, mean, and median ADC values and Gleason scores of 6, 7 and 8 and found the strongest correlation between the 10th percentile ADC values and Gleason score. Given that histogram parameters may reflect tumor heterogeneity, it is of clinical relevance to know whether histogram analysis can serve as a biomarker for the prediction of BCR and whether ADC parameters have an incremental prognostic value in patients with PCa receiving RP and AHT.

To our knowledge, the predictive effect of ADC histogram analysis on the prognosis of high-risk PCa patients receiving AHT after RP remains unclear. Therefore, the purpose of this study was to evaluate the prognostic value of preoperative ADC histogram analysis in this certain population and to develop a prognostic model combining clinical factors and ADC histogram parameters.

## 2 Materials and methods

### 2.1 Patients

The study was approved by our institutional review board, and the requirement for written informed consent was waived because of the retrospective nature. In this study, 88 patients with PCa who received RP and extended pelvic lymph node dissection (ePLND) in our hospital from March 2013 to October 2015 were enrolled. The inclusion criteria were as follows: 1) age ≥18 years; 2) pathologically confirmed prostate adenocarcinoma; 3) high-risk PCa (preoperative serum PSA ≥ 20 ng/mL or Gleason score ≥ 8) or locally advanced PCa (pT3/4, N0M0 or any T, N1M0) or positive surgical margin (R1) (19); 4) preoperative MRI scans, including diffusion-weighted imaging with a b value of 0 and 1000 sec/mm^2^, were performed within 3 months before surgery; and 5) AHT was given immediately after surgery. Of these, 26 males were excluded: initially received additional concurrent anticancer therapy (RT, chemotherapy) before MRI scanning (n = 13), lack of DW images (n = 2), the tumor location was not clearly defined (n = 3), and poor image quality due to severe motion artifacts or susceptibility artifacts (n = 8). Finally, 62 consecutive males were included in this study. All enrolled patients received AHT, including medical castration (LHRHa), combined with anti-androgens (bicalutamide, etc.) immediately after RP. The baseline clinicopathologic data were collected retrospectively from our medical database, including age at RP, preoperative total PSA (TPSA), pathological Gleason score, pathological T and N stages (pT, pN), and surgical margin status (SM R1/R0).

### 2.2 MRI protocol

All patients underwent MRI examinations using a 3.0 T MRI scanner (MAGNETOM Skyra, Siemens Healthcare, Erlangen, Germany) with an 18-element body coil above the pelvis. The MRI sequences included: 1) axial T2-weighted image (repetition time/echo time [TR/TE] 6500-6874/104 msec, slice thickness 3 mm, slice spacing 3 mm, field of view 180 mm×180 mm, matrix 384×346 mm, flip angle 160°, NEX=2; 2) DWI (b = 0, 200, 400, 800,1000, 1500 sec/mm2) was acquired in the axial plane by single-shot echo-planar sequence with the following parameters: TR 4500 ms, TE 85 ms, slice thickness of 3 mm, slice spacing 0 mm, field of view of 214×171 mm, matrix 90 × 72.

### 2.3 Image postprocessing and analysis

**Figure 1:**
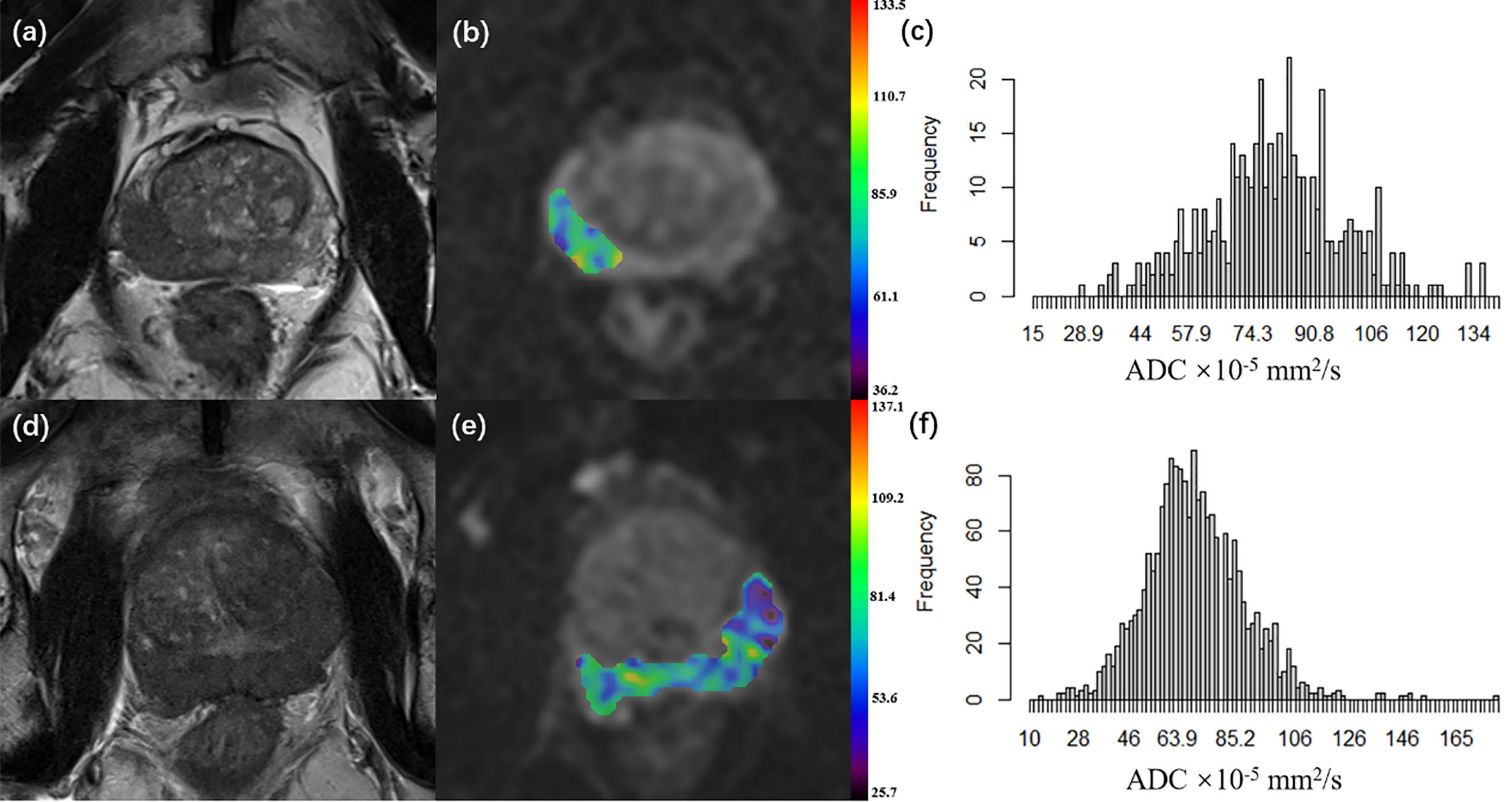
**(A–C)** A 66-year-old patient with pT2 prostate cancer who did not undergo disease progression. **(D–F)** A 68-year-old patient with pT3a prostate cancer who underwent disease progression 5.3 months after surgery. **(A, D)** Axial T2WI; **(B, E)** Axial diffusion-weighted imaging at b 1000 value with ADC map of the lesion VOI generated from b1000 and b0; **(C, F)** ADC histogram generated from the VOI. (pT, pathological T grade; T2WI, T2 weighted imaging; ADC, apparent diffusion coefficient, ADC values are given in units of ×10^-5^ mm^2^/s; VOI, the volume of interest).

All the image data were saved in Digital Imaging and Communication in Medicine (DICOM) format in a personal computer and postprocessed offline with custom-developed software (Firevoxel, https://firevoxel.org). A radiologist (MXY) with more than 5 years of experience in prostate MRI interpretation performed the segmentation of lesions independently and was blinded to the patients’ clinical characteristics and survival outcomes. For each patient, a 3D volume of interest (3D VOI) was manually delineated along the margins of the whole lesion on all continuous sections of DWI (b=1000 s/mm^2^), avoiding the areas of hemorrhage and adjacent nonneoplastic structures, with reference to T2-weighted images. To evaluate the reproducibility of segmentation, another senior radiology resident (LP) with 3 years of prostate mp-MRI experience randomly selected 30 prostate cancers for segmentation. The ADC map within the 3D VOI was calculated automatically by DWIs at b = 0 and 1000 s/mm^2^ (18) using a monoexponential model:


$$\frac{s}{s_{0}}={\text{e}}^{\left(-b\times ADC\right)}$$

where *S* represents the signal intensity at a certain *b*-value and *S_0_* represents the signal intensity at *b*-value = 0 s/mm^2^. The ADC histogram was automatically generated on a voxel-by-voxel basis along with the following corresponding parameters: mean ADC (ADC_mean_), median ADC (ADC_50_), 10th percentile ADC (ADC_10_), 90th percentile ADC (ADC_90_), skewness, and kurtosis, which were the most commonly used histogram parameters in the previous studies of PCa and other cancers. Two typical examples of PCa cases are provided in **Figure 1**.

### 2.4 Clinical outcome

The clinical outcome was biochemical recurrence-free survival (BCR-fs), defined as the time from surgery to biochemical recurrence (BCR). BCR was defined as a PSA increase for two consecutive measurements: a PSA level ≥ 0.2 ng/ml for localized disease and an increase in the PSA level by 25% or more above the nadir (and by ≥2 ng/ml), with confirmation four or more weeks later for lymphatic or distant metastasis cases, according to PCWG3 criteria (20). One patient was censored on the date of the last follow-up.

### 2.5 Statistical analysis

The statistical analysis was performed using R (Version 4.1.2, R Foundation for Statistical Computing, Vienna, Austria) and Stata software package version 12.1 (StataCorp, College Station, Tex). A two-tailed p<0.05 was considered statistically significant. Continuous data are presented as medians and interquartile ranges (IQRs), while categorical data are presented as frequencies and proportions. The Kaplan−Meier method and the log-rank test were used for univariate survival analysis. Continuous variables such as the ADC parameters and age were dichotomized before Kaplan−Meier curve construction by using optimal cutoff values determined by the “surv_cutpoint” function of the “survminer” R package. The factors with *p*< 0.100 in univariate analysis were included in multivariate Cox regression analysis to establish the prognostic model of BCR-fs. Prognostic models were built in two ways: one clinical model without ADC parameters and the other clinical-imaging model with ADC parameters as covariates. The variables in the final models were determined by backward stepwise variable selection on the basis of *p* = 0.100, indicating a significant difference, which can also be used to eliminate multicollinearity. The Harrell concordance index (c index) was calculated to evaluate the performance of the model. In addition, the method mentioned by Newson (21) was used to compare the C indices between models with and without ADC parameters to assess the incremental prognostic value of the ADC parameters. Additionally, receiver operating characteristic curve (ROC) analysis was used to assess the diagnostic accuracy of BCR-fs at 12, 24 and 36 months and to compare the area under the receiver operating characteristic curves (AUC) between the clinical model and clinical imaging model.

Intraclass correlation coefficients (ICCs) were used to evaluate the interrater reliability of ADC parameters. The ICC was interpreted as follows: ≥0.80, excellent reproducibility; 0.61-0.80, good reproducibility; 0.41-0.60, moderate reproducibility; 0.21-0.40, fair reproducibility; and<0.20, poor reproducibility.

## 3 Result

A total of 62 patients (median age: 66.50 years and IQR: 61.75-71.00 years) with pathologically confirmed prostate cancer were included. All patients were followed up for more than 3 years with a median follow-up period of 54.1 months (range, 41.1–79.3 months). BCR was found in 19 patients (30.6%). The BCR-fs rates at 12, 24 and 36 months were 85.5%, 79.0%, and 71.0%, respectively. The baseline characteristics of the enrolled patients are shown in **Table 1**.

**Table 1:** Baseline characteristics of all patients (n=62).

Variables	Median (IQR) or No (%)
Age at surgery (years)	66.50 (61.75-71.00)
Gleason scores	
≤ 7	37(59.68)
> 7	25(40.32)
pT	
2	29(46.77)
3~4	33(53.23)
pN	
0	51(82.26)
1	11(17.74)
Preoperative TPSA (ng/ml)	20.58 (7.87-41.45)
SM	
R0	40(64.52)
R1	22(35.48)
Follow-up time (months)	54.10 (48.07-62.90)

IQR, Inter quartile range; pT, pathological T grade; pN, pathological N grade; TPSA, Total prostate-specific antigen; SM, surgical margin.

ADC histogram parameters demonstrated good to excellent reproducibility (ADC_10_, ICC: 0.892; ADC_90_, ICC: 0.917; ADC_mean_, ICC: 0.803; ADC_50_, ICC: 0.931; kurtosis, ICC: 0.867; skewness, ICC: 0.820).

**Figure 2:**
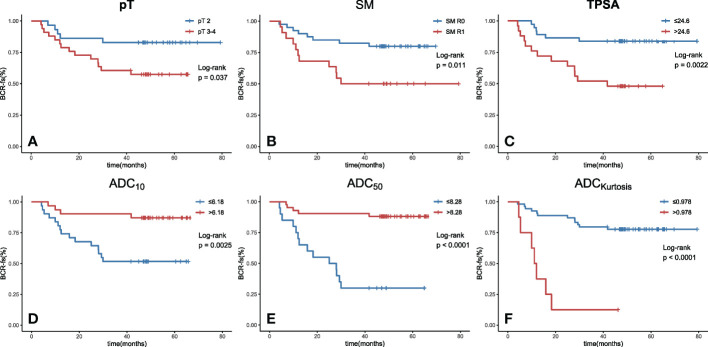
Kaplan−Meier curves with log-rank test for BCR-fs based on **(A)** pT, **(B)** SM, **(C)** TPSA, **(D)** ADC_10_, **(E)** ADC_50_ and **(F)** ADCkurtosis. All of these parameters were significant prognostic factors. (BCR-fs, biochemical recurrence-free survival; pT, pathological T staging; SM, surgical margin; TPSA, total prostate-specific antigen; ADC, apparent diffusion coefficient; the values of ADC^10^, ADC_50_ are given in units of ×10^-4^ mm^2^/s, the value of kurtosis is given in unit of ×10^-1^ and the value of TPSA is given in unit of ×10^-1^ ng/ml.).

In the univariate survival analysis, lower ADC values (ADC_mean_ ≤ 8.59 × 10^−4^ mm^2^/s, ADC_10_ ≤ 6.18 × 10^−4^ mm^2^/s, ADC_50_ ≤ 8.28 × 10^−4^ mm^2^/s, ADC_90_ ≤ 11.7 × 10^−4^ mm^2^/s) and higher kurtosis (> 9.78×10^−1^) were significant predictors of poorer BCR-fs (all *p*<.05, **Table 2**). For clinical parameters, SM R1, TPSA ≥ 24.6 ng/ml, and pT3~pT4 had significant relationships with poor BCR-fs (*p* = 0.011, *p* = 0.002, and *p* = 0.037, respectively). BCR-fs showed no significant differences in age, Gleason score, and pN (**Table 2**). Kaplan−Meier curves of pT, SM, TPSA ADC_10_, ADC_50_, and ADC_kurtosis_ for BCR-fs are shown in **Figure 2**.

**Table 2:** Univariable analysis of BCR-fs by K-M analysis and log-rank test.

Variables	*p* value
Age at surgery (ref: ≤72 years)	0.222
Preoperative TPSA (ref: ≤24.6 ng/ml)	**0.002**
Gleason scores (ref: ≤7)	0.427
pT3~4 (ref: pT2)	**0.037**
pN1 (ref: pN0)	0.541
SM R1 (ref: R0)	**0.011**
ADC_mean_ (ref: ≤8.59 × 10^−4^ mm^2^/s)	**<0.001**
ADC_10_ (ref: ≤6.18× 10^−4^ mm^2^/s)	**0.003**
ADC_50_ (ref: ≤8.28× 10^−4^ mm^2^/s)	**<0.001**
ADC_90_ (ref: ≤11.7× 10^−4^ mm^2^/s)	**0.001**
Skewness (ref: ≤7.80)	0.318
Kurtosis (ref: ≤9.78)	**<0.001**

BCR-fs, biochemical recurrence-free survival; TPSA, total prostate-specific antigen; pT, pathological T grade; pN, pathological N grade; SM, surgical margin. p values were calculated from the log-rank test. p values in bold denote statistical significance.

Multivariate Cox regression analysis was performed for ADC_mean_, ADC_10_, ADC_50_, ADC_90_, and kurtosis histogram parameters, as well as preoperative TPSA, pT and SM status. ADC_50_ and kurtosis remained significant (HR: 0.172, 95% CI: 0.055-0.541, *p* = 0.003; HR: 7.058, 95% CI: 2.288-21.773, *p* = 0.001;, respectively) after adjustment for clinical factors. The clinical parameters of preoperative TPSA and SM were independent predictors of BCR-fs (HR = 3.824, 95% CI = 1.406-10.402, *p* = 0.009; HR = 3.067, 95% CI = 1.110-8.477, *p* = 0.031, respectively).

**Figure 3:**
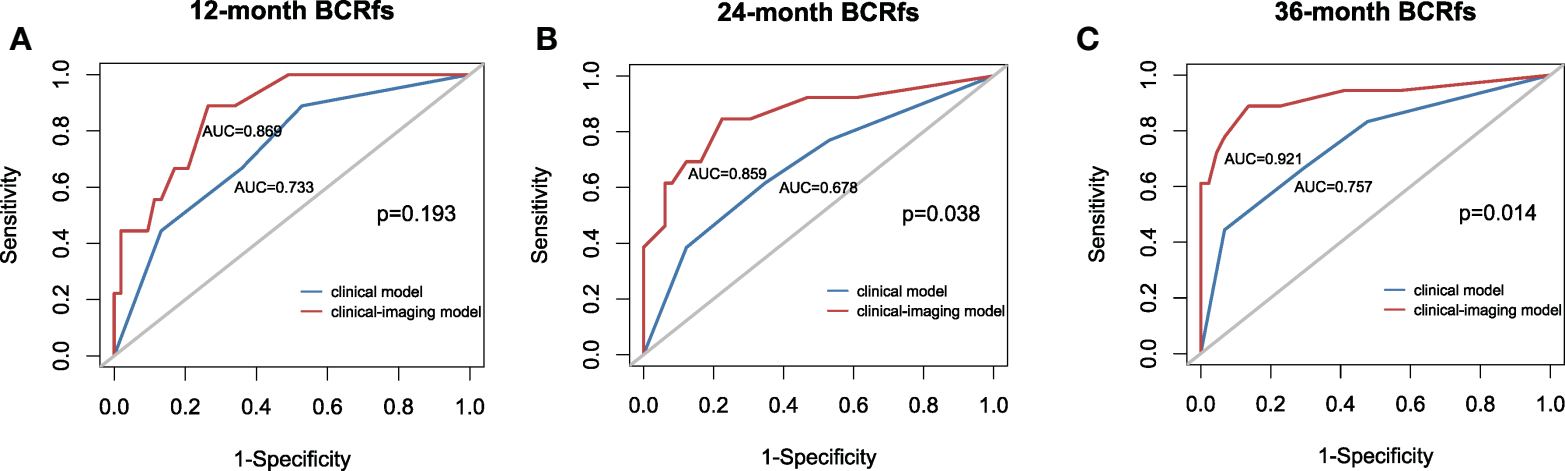
Receiver operating characteristic (ROC) curve with the clinical model and clinical-imaging model used for predicting the **(A)** 12-month BCR-fs, **(B)** 24-month BCR-fs and **(C)** 36-month BCR-fs. The clinical imaging model had higher AUCs than the clinical model in predicting the 12-month, 24-month and 36-month BCR-fs. AUC, area under the curve; BCR-fs, biochemical recurrence-free survival.

Then, the clinical model was established with TPSA and surgical margins, while the clinical imaging model was established with ADC_50_ and kurtosis as covariables. All variables in the clinical model remained significant predictors in multivariate prognostic models of BCR-fs (*p*<.100 for all), as shown in **Table 3**. In the comparison of model performance, the C index of the clinical-imaging model was higher than that of the clinical model (C index = 0.85, 95% CI: 0.76-0.94; C index = 0.73, 95% CI: 0.61-0.84, respectively, *p* = 0.032). The inclusion of histogram parameters improved the diagnostic accuracy of the models for 12-, 24- and 36-month BCR-fs (AUC of clinical imaging model vs clinical model: 0.869 VS 0.733, *p* = 0.19; 0.859 VS 0.678, *p* = 0.038; and 0.921 VS 0.7557, *p* = 0.014; for 12-month, 24-month and 36-month BCR-fs, respectively), in which the improvement in the diagnostic accuracy of BCR-fs at 24 and 36 months was statistically significant (**Figure 3**).

**Table 3:** Prognostic model analysis with and without histogram parameters for BCR-fs.

	Clinical model			Clinical-imaging model		
	HR	95% CI	*p* value	HR	95% CI	*p* value
TPSA	3.776	1.426-9.994	0.007	3.824	1.406-10.402	0.009
SM	2.822	1.126-7.074	0.027	3.067	1.11-8.477	0.031
ADC_50_	/	/	/	0.172	0.055-0.541	0.003
ADC_kurtosis_	/	/	/	7.058	2.288-21.773	0.001

BCR-fs, biochemical recurrence-free survival; TPSA, total prostate-specific antigen; SM, surgical margin; ADC, apparent diffusion coefficient; HR, hazard ratio; CI, confidence interval.

## 4 Discussion

In this study, we evaluated the prognostic value of the ADC histogram derived from preoperative DWI in predicting the BCR of patients with high-risk PCa who underwent RP followed by AHT. The results showed that the ADC_50_ and kurtosis of the ADC histogram were independent prognostic factors for BCR-fs, and when ADC parameters were incorporated into the clinical model, the prediction accuracy for BCR-fs was improved.

Several tools have been proposed to identify patients at risk for BCR, including preoperative tools, such as the Kattan Nomogram (22) and Prostate Cancer Risk Assessment (CAPRA) score (23), and postoperative tools, such as the Postsurgery Prostate Cancer Risk Assessment (CAPRA-S) score (24, 25). These assessments showed that factors including PSA and SM status were significantly associated with the risk of BCR (24). In our study, we also found that preoperative TPSA, pT stage and SM status were associated with the risk of BCR. Although most studies have demonstrated that the Gleason score is a risk factor for BCR, our study did not find a significant association between the Gleason score and the risk of BCR, similar to the results from a prospective, multicenter cohort of patients with high-risk localized and locally advanced prostate cancer in China who also received adjuvant hormone therapy after radical prostatectomy (26). Moreover, the BCR rate at the 24-month follow-up in our study was 21%, which is also similar to the BCR rate (17.4% at the 24-month follow-up) in that prospective study (26). We did not find any relationship between pN stage and BCR. The differences in the results between our study and other studies may be because of the heterogeneity of the study population and the limited sample size and follow-up time.

MRI is an essential clinical examination for prostate cancer. The diffusion-weighted imaging of MRI is a functional sequence that reflects the degree of restricted diffusion of water and is thought to be related to the cellularity and interstitial structure of pathological tissue. Tumors with different biological behaviors, such as metastasis and recurrence potential, may show different degrees of diffusion restriction. Attempts have been made to integrate MRI into prognostic models to improve the predictive performance (9, 10). Haralick features on T2WI have been previously shown to be associated with BCR in PCa (27, 28). However, the information embodied in the T1 and T2 sequences is more anatomical than functional, and not all patients underwent dynamic contrast-enhanced examination, so we chose DWI as the research object, similar to other histogram analysis studies of different cancers. Bourbonne et al. (29) used radiomics to analyze BCR and BCR-free survival in 107 high-risk PCa patients (pT3–pT4, positive margins, GS 8–10) treated with RP. They found that a texture feature, small zone emphasis (SZE_GLSZM_) from the ADC map, was strongly correlated with the risk of BCR, independent of other radiomic features and clinical variables. However, the study reported that the MRI was performed on two different MRI scanners: a Phillips 3T and a Siemens 1.5T, which could have some relevant impact on the results. Compared to high-order texture features, which may involve complex statistical computations and less interpretability, first-order textural analysis can be better understood and may have more clinical validity (30).

ADC histogram analysis can yield additional parameters, such as different percentiles of ADC values, skewness, and kurtosis, which can provide more diffusion information than qualitative evaluation and can better reflect tumor heterogeneity. In this study, we evaluated percentile values of ADC_10_ and ADC_90_, because they were far from the ADC_mean_, may provide information that the ADC_mean_ cannot, and are less susceptible to extreme values, like ADC_max_ and ADC_min_. Rosenkrantz et al. (31) found that ADC_0-10_, the mean of the bottom 10th percentile ADC, rather than the average value of the ADC, was a significant independent predictor for BCR in the multivariate logistic regression analysis. Donati et al. (18) also observed that the AUC of the 10^th^ percentile ADC was significantly higher than the mean ADC to predict the Gleason score. They argued that the 10^th^ percentile ADC was more representative of focal areas of high cellularity than the mean or median values, which were more susceptible to normal glandular tissue (18). However, according to our results, ADC_50_ remained a significant parameter after stepwise backward regression to rule out collinearity of other ADC percentile values, which means that ADC_50_ predicts BCR-fs better than ADC_10_ in PCa patients after RP and AHT. While the reason remains unclear, one possible explanation for the difference between the results of this study and the previous ones is that we studied a different population. We enrolled patients with high-risk localized or locally advanced PCa whose cancer foci may be more prone to necrosis and hemorrhage. When delineating the ROI, micronecrosis and microhemorrhage lesions within the tumor were inevitable, leading to increased ADC values. We assume that micronecrosis and microhemorrhage also play a role in the prognostic value of the ADC histogram; thus, higher percentile ADCs may more comprehensively predict the outcome than lower percentile ADCs. These controversial results indicated the need for further investigation of the mechanisms underlying the association between different percentile ADCs and prognostic values.

Kurtosis is an important indicator of tumor homogeneity and has been reported to be associated with the prognosis of several cancers. Higher kurtosis on DWI was associated with poorer overall survival and relapse-free survival in surgically treated colorectal cancer patients (32). In thyroid cancer, ADC kurtosis was associated with the aggressiveness of the lesion, and higher kurtosis predicted lymphatic metastasis (33). In addition, kurtosis of the ADC histogram could serve as a potential biomarker for the prediction of tumor aggressiveness and survival in esophageal cancer (15). We speculate that the profound changes in the microarchitecture of distinct tumor cell clusters may be related to their recurrence potential, which is reflected in the corresponding changes in the ADC histogram.

In this study, we hypothesized that the prognostic value might increase when ADC histogram parameters were added to traditional clinical factors. Further comparison of the performance of the clinical imaging model and clinical model showed that the C index increased and the *p* value was 0.032. We also observed an increase in the AUC of the diagnostic accuracy of BCR-fs at 24 months and 36 months. This trend leads us to believe that ADC histogram analysis has incremental prognostic value in the prognosis of PCa patients.

This study has several limitations. First, this single-center, retrospective study could have some possible bias, which needs to be validated in multicenter prospective studies to provide more reliable evidence for clinical application. Second, the sample size is small, with only 62 patients’ survival information available, and it is difficult to set aside an independent validation cohort, which may not have sufficient power to draw conclusions. An independent cohort to validate this finding is required in the future. Studies with larger sample sizes may produce more accurate predictions. In addition, the follow-up time was relatively short, with longer follow-up times required to predict 5-year and 10-year BCR-fs, progression-free survival and overall survival. Besides, the delineation of the tumor ROIs was subjectively free-hand, which may reduce the research reproducibility. A standardization and consensus of the DWI protocol, including the imaging postprocessing method, especially automatic or semiautomatic segmentation, are warranted. Last but not least, this prostate prognostic model was constructed based on high-risk localized and locally advanced PCa, the community-based benefits of this model are limited.

## 5 Conclusion

Preoperative MRI-derived ADC histogram analysis has incremental prognostic value as an imaging biomarker for the prognosis of patients with high-risk PCa receiving AHT after RP. Our study results demonstrate that ADC_50_ and kurtosis are the most valuable histogram-derived parameters for predicting BCR-fs in high-risk PCa patients.

## Data availability statement

The raw data supporting the conclusions of this article will be made available by the authors, without undue reservation.

## Ethics statement

The studies involving human participants were reviewed and approved by the Tongji Hospital of Huazhong University of Science and Technology (Wuhan, China) ethics review committee. Written informed consent for participation was not required for this study in accordance with the national legislation and the institutional requirements.

## Author contributions

XM and HL had full access to all the data used in this study and takes responsibility for the accuracy of the data analysis. KH and YZ contributed equally to this work. Study concept and design, XM and HL. Drafting of the manuscript, KH and YZ. Critical revision of the manuscript for important intellectual content, all authors. Image obtaining and postprocessing, XM and PL. Statistical analysis, SL, GY, and QZ. Administrative, technical, or material support, HL and ZL. Study supervision, ZL. All authors contributed to the article and approved the submitted version.

## References

[1] Sung H Ferlay J Siegel RL Laversanne M Soerjomataram I Jemal A. Global cancer statistics 2020: GLOBOCAN estimates of incidence and mortality worldwide for 36 cancers in 185 countries. CA Cancer J Clin (2021) 71(3):209–49. doi: 10.3322/caac.21660 33538338

[2] Mottet N Bellmunt J Bolla M Briers E Cumberbatch MG De Santis M. EAU-ESTRO-SIOG guidelines on prostate cancer. part 1: Screening, diagnosis, and local treatment with curative intent. Eur Urol (2017) 71(4):618–29. doi: 10.1016/j.eururo.2016.08.003 27568654

[3] Messing EM Manola J Yao J Kiernan M Crawford D Wilding G. Immediate versus deferred androgen deprivation treatment in patients with node-positive prostate cancer after radical prostatectomy and pelvic lymphadenectomy. Lancet Oncol (2006) 7(6):472–9. doi: 10.1016/s1470-2045(06)70700-8 16750497

[4] Siddiqui SA Boorjian SA Inman B Bagniewski S Bergstralh EJ Blute ML. Timing of androgen deprivation therapy and its impact on survival after radical prostatectomy: A matched cohort study. J Urol (2008) 179(5):1830–7. doi: 10.1016/j.juro.2008.01.022 18353378

[5] Bastide C Rossi D Lechevallier E Bladou F Barriol D Bretheau D. Seminal vesicle invasion: What is the best adjuvant treatment after radical prostatectomy? BJU Int (2012) 109(4):525–30. doi: 10.1111/j.1464-410X.2011.10332.x 21851534

[6] Siddiqui SA Boorjian SA Blute ML Rangel LJ Bergstralh EJ Karnes RJ. Impact of adjuvant androgen deprivation therapy after radical prostatectomy on the survival of patients with pathological T3b prostate cancer. BJU Int (2011) 107(3):383–8. doi: 10.1111/j.1464-410X.2010.09565.x 21265985

[7] Chang AJ Autio KA Roach M 3rd Scher HI. High-risk prostate cancer-classification and therapy. Nat Rev Clin Oncol (2014) 11(6):308–23. doi: 10.1038/nrclinonc.2014.68 PMC4508854 24840073

[8] Turkbey B Choyke PL. Prostate magnetic resonance imaging: Lesion detection and local staging. Annu Rev Med (2019) 70:451–9. doi: 10.1146/annurev-med-053117-123215 30691375

[9] Park JJ Kim CK Park SY Park BK Lee HM Cho SW. Prostate cancer: Role of pretreatment multiparametric 3-T MRI in predicting biochemical recurrence after radical prostatectomy. AJR Am J roentgenol (2014) 202(5):W459–65. doi: 10.2214/AJR.13.11381 24758681

[10] Hattori S Kosaka T Mizuno R Kanao K Miyajima A Yasumizu Y. Prognostic value of preoperative multiparametric magnetic resonance imaging (MRI) for predicting biochemical recurrence after radical prostatectomy. BJU Int (2014) 113(5):741–7. doi: 10.1111/bju.12329 23937660

[11] Padhani AR Liu G Mu-Koh D Chenevert TL Thoeny HC Takahara T. Diffusion-weighted magnetic resonance imaging as a cancer biomarker: Consensus and recommendations. Neoplasia (2009) 11(2):102–25. doi: 10.1593/neo.81328 PMC2631136 19186405

[12] Le Bihan D. Apparent diffusion coefficient and beyond: What diffusion MR imaging can tell us about tissue structure. Radiology (2013) 268(2):318–22. doi: 10.1148/radiol.13130420 23882093

[13] Zelhof B Pickles M Liney G Gibbs P Rodrigues G Kraus S. Correlation of diffusion-weighted magnetic resonance data with cellularity in prostate cancer. BJU Int (2009) 103(7):883–8. doi: 10.1111/j.1464-410X.2008.08130.x 19007373

[14] Choi YS Ahn SS Kim DW Chang JH Kang SG Kim EH. Incremental prognostic value of ADC histogram analysis over MGMT promoter methylation status in patients with glioblastoma. Radiology (2016) 281(1):175–84. doi: 10.1148/radiol.2016151913 27120357

[15] Hirata A Hayano K Ohira G Imanishi S Hanaoka T Toyozumi T. Volumetric histogram analysis of apparent diffusion coefficient as a biomarker to predict survival of esophageal cancer patients. Ann Surg Oncol (2020) 27(8):3083–9. doi: 10.1245/s10434-020-08270-7 32100222

[16] Zhao B Cao K Li XT Zhu HT Sun YS. Whole lesion histogram analysis of apparent diffusion coefficients on MRI predicts disease-free survival in locally advanced squamous cell cervical cancer after radical chemo-radiotherapy. BMC Cancer (2019) 19(1):1115. doi: 10.1186/s12885-019-6344-3 31729974 PMC6858752

[17] Peng Y Jiang Y Yang C Brown JB Antic T Sethi I. Quantitative analysis of multiparametric prostate MR images: differentiation between prostate cancer and normal tissue and correlation with Gleason score–a computer-aided diagnosis development study. Radiology (2013) 267(3):787–96. doi: 10.1148/radiol.13121454 PMC6940008 23392430

[18] Donati OF Mazaheri Y Afaq A Vargas HA Zheng J Moskowitz CS. Prostate cancer aggressiveness: assessment with whole-lesion histogram analysis of the apparent diffusion coefficient. Radiology (2014) 271(1):143–52. doi: 10.1148/radiol.13130973 24475824

[19] EAU Guidelines. Edn. presented at the EAU annual congress Amsterdam. (2022), ISBN 978-94-92671-16-5. Available at: https://uroweb.org/guidelines/prostate-cancer/chapter/citation-information.

[20] Scher HI Morris MJ Stadler WM Higano C Basch E Fizazi K. Trial design and objectives for castration-resistant prostate cancer: Updated recommendations from the prostate cancer clinical trials working group 3. J Clin Oncol (2016) 34(12):1402–18. doi: 10.1200/JCO.2015.64.2702 PMC4872347 26903579

[21] Newson RB. Comparing the predictive powers of survival models using harrell’s c or somers’ d. STATA J (2010) 10(3):339–58. doi: 10.1177/1536867X1001000303

[22] Kattan MW Eastham JA Stapleton AM Wheeler TM Scardino PT. A preoperative nomogram for disease recurrence following radical prostatectomy for prostate cancer. J Natl Cancer Institute (1998) 90(10):766–71. doi: 10.1093/jnci/90.10.766 9605647

[23] Cooperberg MR Pasta DJ Elkin EP Litwin MS Latini DM Du Chane J. The university of California, San Francisco cancer of the prostate risk assessment score: A straightforward and reliable preoperative predictor of disease recurrence after radical prostatectomy. J Urol (2005) 173(6):1938–42. doi: 10.1097/01.ju.0000158155.33890.e7 PMC2948569 15879786

[24] Cooperberg MR Hilton JF Carroll PR. The CAPRA-s score: A straightforward tool for improved prediction of outcomes after radical prostatectomy. Cancer (2011) 117(22):5039–46. doi: 10.1002/cncr.26169 PMC3170662 21647869

[25] Punnen S Freedland SJ Presti JC Jr. Aronson WJ Terris MK Kane CJ. Multi-institutional validation of the CAPRA-s score to predict disease recurrence and mortality after radical prostatectomy. Eur Urol (2014) 65(6):1171–7. doi: 10.1016/j.eururo.2013.03.058 23587869

[26] Ye D Zhang W Ma L Du C Xie L Huang Y. Adjuvant hormone therapy after radical prostatectomy in high-risk localized and locally advanced prostate cancer: First multicenter, observational study in China. Chin J Cancer Res (2019) 31(3):511–20. doi: 10.21147/j.issn.1000-9604.2019.03.13 PMC6613498 31354220

[27] Gnep K Fargeas A Gutierrez-Carvajal RE Commandeur F Mathieu R Ospina JD. Haralick textural features on T2 -weighted MRI are associated with biochemical recurrence following radiotherapy for peripheral zone prostate cancer. J Magn Reson Imaging (2017) 45(1):103–17. doi: 10.1002/jmri.25335 27345946

[28] Shiradkar R Ghose S Jambor I Taimen P Ettala O Purysko AS. Radiomic features from pretreatment biparametric MRI predict prostate cancer biochemical recurrence: Preliminary findings. J Magn Reson Imaging (2018) 48(6):1626–36. doi: 10.1002/jmri.26178 PMC6222024 29734484

[29] Bourbonne V Vallières M Lucia F Doucet L Visvikis D Tissot V. MRI-Derived radiomics to guide post-operative management for high-risk prostate cancer. Front Oncol (2019) 9:807. doi: 10.3389/fonc.2019.00807 31508367 PMC6719613

[30] Patel N Henry A Scarsbrook A. The value of MR textural analysis in prostate cancer. Clin Radiol (2019) 74(11):876–85. doi: 10.1016/j.crad.2018.11.007 30573283

[31] Rosenkrantz AB Ream JM Nolan P Rusinek H Deng FM Taneja SS. Prostate cancer: Utility of whole-lesion apparent diffusion coefficient metrics for prediction of biochemical recurrence after radical prostatectomy. AJR Am J roentgenol (2015) 205(6):1208–14. doi: 10.2214/AJR.15.14482 PMC4691847 26587927

[32] Takahashi Y Hayano K Ohira G Imanishi S Hanaoka T Watanabe H. Histogram analysis of diffusion-weighted MR imaging as a biomarker to predict survival of surgically treated colorectal cancer patients. Dig Dis Sci (2021) 66(4):1227–32. doi: 10.1007/s10620-020-06318-y 32409951

[33] Schob S Meyer HJ Dieckow J Pervinder B Pazaitis N Höhn AK. Histogram analysis of diffusion weighted imaging at 3T is useful for prediction of lymphatic metastatic spread, proliferative activity, and cellularity in thyroid cancer. Int J Mol Sci (2017) 18(4):821. doi: 10.3390/ijms18040821 28417929 PMC5412405

